# The Phytoestrogen Genistein Affects Zebrafish Development through Two Different Pathways

**DOI:** 10.1371/journal.pone.0004935

**Published:** 2009-03-25

**Authors:** Sana Sassi-Messai, Yann Gibert, Laure Bernard, Shin-Ichi Nishio, Karine F. Ferri Lagneau, José Molina, Monika Andersson-Lendahl, Gérard Benoit, Patrick Balaguer, Vincent Laudet

**Affiliations:** 1 Institut de Génomique Fonctionnelle de Lyon, Université de Lyon, Université Lyon 1, CNRS, INRA, Ecole Normale Supérieure de Lyon, Lyon, France; 2 Apoptosis and Oncogenesis Laboratory, Institut de Biologie et Chimie des Protéines, Université de Lyon, Université Lyon 1, Institut Fédératif Biosciences Gerland Lyon Sud, CNRS, Lyon, France; 3 Karolinska Institutet, Department of Biosciences and Nutrition, Laboratory of Medical Nutrition, Stockholm, Sweden; 4 Equipe INSERM U896 Institut de Recherche en Cancérologie de Montpellier (IRCM), Montpellier, France; Institute of Genetics and Molecular and Cellular Biology, France

## Abstract

**Background:**

Endocrine disrupting chemicals are widely distributed in the environment and derive from many different human activities or can also be natural products synthesized by plants or microorganisms. The phytoestrogen, genistein (4′, 5, 7-trihydroxy-isoflavone), is a naturally occurring compound found in soy products. Genistein has been the subject of numerous studies because of its known estrogenic activity.

**Methodology/Principal Findings:**

We report that genistein exposure of zebrafish embryos induces apoptosis, mainly in the hindbrain and the anterior spinal cord. Timing experiments demonstrate that apoptosis is induced during a precise developmental window. Since adding ICI 182,780, an ER antagonist, does not rescue the genistein-induced apoptosis and since there is no synergistic effect between genistein and estradiol, we conclude that this apoptotic effect elicited by genistein is estrogen-receptors independent. However, we show *in vitro*, that genistein binds and activates the three zebrafish estrogen receptors ERα, ERβ-A and ERβ-B. Furthermore using transgenic ERE-Luciferase fish we show that genistein is able to activate the estrogen pathway *in vivo* during larval stages. Finally we show that genistein is able to induce ectopic expression of the *aromatase-B* gene in an ER-dependent manner in the anterior brain in pattern highly similar to the one resulting from estrogen treatment at low concentration.

**Conclusion/Significance:**

Taken together these results indicate that genistein acts through at least two different pathways in zebrafish embryos: (i) it induces apoptosis in an ER-independent manner and (ii) it regulates *aromatase-B* expression in the brain in an ER-dependent manner. Our results thus highlight the multiplicity of possible actions of phytoestrogens, such as genistein. This suggests that the use of standardized endpoints to study the effect of a given compound, even when this compound has well known targets, may carry the risk of overlooking interesting effects of this compound.

## Introduction

There is considerable concern over the environmental occurrence of endocrine-disrupting chemicals (EDCs), i.e., natural and man-made substances that interfere with the endocrine system of vertebrates [1], [2], [3]. These molecules have the potential to modulate or disrupt the synthesis, secretion, transport, binding, action, or elimination of endogenous hormones in the body and consequently to affect homeostasis, development, reproduction, and behavior in many organisms, including humans. EDCs are now widely distributed in the environment and derive from many different human activities (pesticides, industrial products, cancer drugs, fattening agents) and are also found as natural products (human and animal waste; phytoestrogens).

Genistein (4′,5,7-trihydroxy-isoflavone) belongs to the isoflavone class of flavonoids and is classified as a phytoestrogen, i.e., plant-derived compounds that possess estrogen-like biological activity (reviewed in [4], [5], [6]). Genistein, found in various soy foods at concentrations of 1.9–229 µg/g, is the main component of soy implicated in cancer chemoprevention [7] and is being evaluated in preliminary trials for breast, prostate and uterus cancers treatment [8], [9], in osteoporosis [10], cardiovascular diseases [11] and for treatment of menopausal symptoms [12].

Genistein is known to bind the human estrogen receptors (ERs) and exhibits estrogenic activities [5], [13], [14], [15]. The actions of estrogens, antiestrogens, and phytoestrogens are mediated by two different estrogen receptors, ERα and ERβ whose expression levels vary dramatically among different organs or cell types (see review in [16], [17]). The two receptors regulate different sets of biological functions and induce dissimilar responses within the same cell type or tissue (reviewed in [16]). Interestingly, it has been shown that genistein preferentially binds human ERβ over the human ERα: compared with ERα, ERβ exhibits a 7–70-fold greater binding affinity for genistein, whereas E2 (17β-estradiol) binds ERα and ERβ with equal affinity [13], [18], [19]. Recent detailed comparison of isoflavones such as genistein and the natural estrogen E2 suggest that their ERβ selectivity involves a capacity to induce an activation function-2 (AF-2) surface of ERβ that has greater affinity for coregulators such as glucocorticoid interacting receptor protein 1 compared with ERα. ([20]; see review in [21]). Therefore, it is now believed that phytoestrogens like genistein, act as natural selective estrogen receptor modulators (SERMs) that elicit distinct clinical effects from estrogens by selectively recruiting coregulatory proteins to ERβ triggering specific transcriptional pathways ([20]; see a review on selective agonists of nuclear receptor ligands in [22]). These functional data have been fully confirmed and extended by detailed structural analysis of human ERα and ERβ complex with genistein [23], [24].

Given this known mode of action, genistein has been the subject of numerous studies in experimental animals and humans because of its possible beneficial and adverse health effects due to estrogenic activity [21], [25]. Recently, several other targets of genistein have been identified suggesting that the mode of action of this dietary compound is more complex than previously expected. For example it has been suggested that genistein acting in the micromolar range is able to bind to transthyretin and can have a beneficial effect as an inhibitor of transthyretin amyloidosis [26]. One of the first described effects of genistein, which was published over two decades ago, is the *in vitro* inhibition at the nanomolar range of the tyrosine-specific protein kinase activity of the epidermal growth factor (EGF) receptor pp60^v-arc^ and pp110^gag-fes^ [27]. Moreover, genistein at 50 µM was shown to be an inhibitor of cyclic AMP-phosphodiesterase (cAMP-PDE) activity resulting in an increased cAMP accumulation [28]. Thus some of the genistein targets have already been identified but its effects during vertebrate embryonic development are still poorly defined. In addition, the relative effects of these various pathways *in vivo* remain to be determined.

Throughout the last decade, several fish species have been considered as potential test organisms for evaluating the endocrine disrupting properties of natural and synthetic chemicals in *in vivo* test systems [29], [30], [31]. Since the first original description [32], numerous reports have emphasized that the effluents of sewage treatment plants induced sexual abnormalities, such as a high incidence of intersex animals in various fish species [33]. Interestingly, recent studies have shown that isoflavone compounds, including genistein, are discharged in water effluents and are present in agricultural runoff as a result from intensive livestock management. For instance, genistein was identified at a concentration of 10 µg/L in the effluents from a pulp mill in Ontario, Canada [34]. There have been relatively few studies on the effects of genistein in teleosts fish and most of these studies were focused on its estrogenic activity. Injections of genistein into sturgeons (*Acipenser baeri*) induce vitellogenesis, a typical estrogenic response in fish [35]. When genistein was intraperitonally injected into the Japanese medaka (*Oryzias latipes*), the plasma E2 levels were increased in exposed females and plasma testosterone levels were reduced in exposed males [36]. In addition, exposure to genistein at 1 mg/L causes gonadal intersex in male medakas as well as delayed oocyte maturation, atretic oocytes and enlarged ovarian lumen in the female [37]. Gametogenesis and reproductive efficiency were reduced in rainbow trout (*Oncorhynchus mykiss*) exposed to genistein-enriched diets for one year [38].

All these studies were mainly focused on sexual abnormalities of the treated fish, because of the known estrogen effects (and by extension phytoestrogens such as genistein) in sex determination and gonad differentiation. Strikingly, however, only very few studies have looked at the developmental defects induced by genistein exposure in fish.

The zebrafish (*Danio rerio*) has become one of the most popular and well-established models in genetics and developmental biology [39], [40], [41]. In addition the zebrafish is commonly used in toxicology to study effects of pollutants such as endocrine disruptors [42], [43] and as a model organism in drug discovery [44]. This model is valuable because of its high reproductive capacity and the transparency of the embryos as well as its rapid development that allow morphological observations of the organogenesis after pharmacological treatments.

In the present study, we have studied the effects induced by genistein exposure during zebrafish embryonic development. We found that genistein treatment induces apoptosis mainly in the hindbrain and in the anterior spinal cord in early zebrafish embryos. We observed that this effect was not impaired in the presence of the anti-estrogen ICI 182,780 suggesting that it is induced through an ER-independent pathway. In contrast, we found that genistein can indeed act through the estrogen receptors both *in vitro* and *in vivo*. Using transgenic ERE-luciferase fish, we demonstrate that genistein activates the estrogen pathway, *in vivo*. Finally, we found that genistein is able to induce ectopic expression of the *aromatase-B* gene in the brain in an ER-dependent manner. We conclude that genistein has two separate effects *in vivo* during zebrafish embryogenesis: (i) inducing early apoptosis in an ER-independent manner and (ii) regulating specific gene expression in an ER-dependent manner. Our results thus highlight the complex *in vivo* effects that can be elicited by a single EDC molecule.

## Results

### Genistein induces apoptosis during zebrafish embryonic development

In order to determine the phenotypic abnormalities induced by genistein exposure during zebrafish embryonic development, we treated zebrafish embryos at concentrations up to 17.5 µM, after the mid-blastula transition when transcription of the zygotic genome first occurs [45]. We observed that at high concentration (17.5 µM) genistein induces a strong mortality. At this concentration, 90% of the embryos survive up to 96 hours post fertilization (hpf), but mortality increases dramatically afterwards and only 35% are still alive at 105 hpf, the few remaining embryos dying shortly after this time point. The survival curve (data not shown) indicates that treatments at 10 µM genistein or below do not induce death of the embryos prior to 105 hpf (larval development was not monitored).

**Figure 1:**
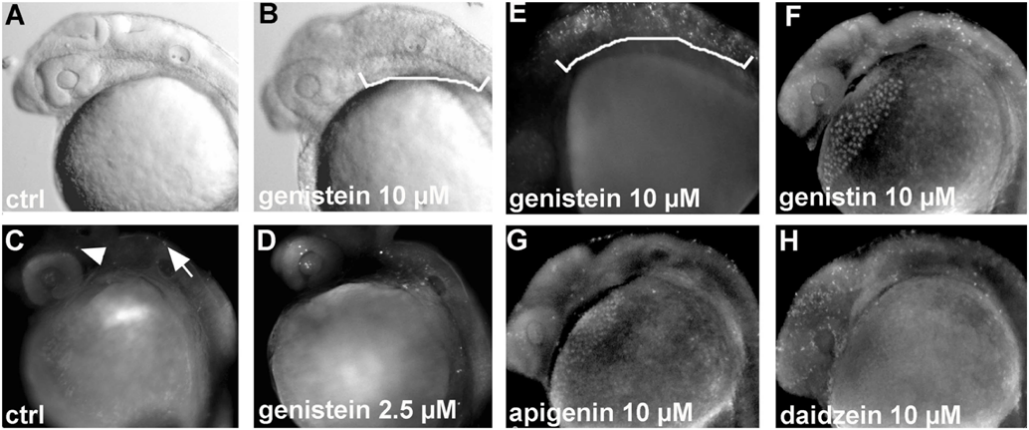
Genistein induces apoptosis during zebrafish development (A) 24 hpf photo of an untreated embryo and an embryo treated with 10 µM of genistein showing necrosis in the posterior hindbrain and in the anterior spinal cord marked by the white bracket (B). (C–H) Apoptotic cell detection at 24 hpf using acridine orange. (C) Control embryo with few apoptotic cells in the midbrain (arrowhead) and the hindbrain (arrow). (D, E) Embryo exposed to 2.5 and 10 µM of genistein showing an increased number of stained cells especially in the hindbrain and a high number of apoptotic cells in the hindbrain and anterior spinal cord when high concentrations of genistein are used (bracket in F). (F–H) Apoptosis mainly found in the hindbrain (arrows) induced by compounds related to genistein: genistin (F), apigenin (G) and daidzein (H) each at 10 µM. Using daidzein, numerous apoptotic cells are detected in the forebrain in comparison to what is observed with the other compounds.

We observed that the dying embryos presented obvious areas of dying cells located in the neural tube, posterior to the mid-hindbrain boundary (MHB) (compare Figure 1A with bracket in Figure 1B). The number of dying cells increases with the concentration of genistein used (data not shown). To determine if these cells were apoptotic, we stained genistein treated embryos at 24 hpf with acridine orange that specifically marks the apoptotic cells [46]. Few apoptotic cells are detected in control embryos at the level of the MHB (arrowhead in Figure 1C) and in the dorsal hindbrain (arrow in Figure 1C), whereas we found a lot more apoptotic cells in genistein treated embryos (Figure 1D, E). We observed that the number of acridine orange positive cells increases in a dose-dependent manner when the embryos are treated with genistein from 2.5 µM to 10 µM (compared Figure 1D with 1E). The acridine orange staining confirms the localization of apoptotic cells mainly in the hindbrain and the anterior spinal cord (bracket in Figure 1E). Interestingly, we did not detect apoptotic cells outside the central nervous system (CNS).

We also treated zebrafish embryos with other isoflavones related to genistein, namely genistin (the glycosylated form of genistein found in soy products accounting to >65% of the isoflavone content), apigenin (one of the citrus bioflavonoids) and daidzein (found widely in plants particularly in soybeans). We found that treatments of zebrafish embryos with 10 µM of these compounds also induce apoptosis (Figure 1. F–H). Of note, daidzein exposure induces apoptosis in a more anterior domain, mainly in the forebrain and midbrain (Figure 1H), which contrasts with what is observed when using the other compounds. Thus, it appears that a large spectrum of isoflavonoids can induce apoptosis in early zebrafish embryos. If we compare the efficiency of all of these compound, we found that at 10 µM genistein exposure leads to a maximal number of apoptotic cells, followed by daidzein and genistin, respectively. Among the tested compounds apigenin is the flavonoids that induces the fewest number of apoptotic cells.

We next confirmed the apoptotic status of the acridine orange labelled cells using TUNEL assays, a direct method detecting the presence of fragmented DNA [47]. Observation of fish labelled at 24 hpf by TUNEL assay confirms that genistein induces apoptosis in a dose-dependent manner (see Figure S1 available in the web). Moreover, using TUNEL assays that are more sensitive than acridine orange staining we found that genistein induces apoptosis at a concentration as low as 0.5 µM. A concentration of genistein of 0.1 µM does not lead to a higher number of apoptotic cells than in control embryos.

Altogether these results demonstrate that genistein exposure induces significant cell death in the hindbrain and anterior spinal cord during zebrafish embryonic development in a dose-dependent manner. Interestingly, the effect is visible at relatively low doses of genistein, since we detect increased TUNEL staining at 24 hpf in embryos treated with 0.5 µM genistein.

### Apoptosis is induced during early developmental stages

**Figure 2:**
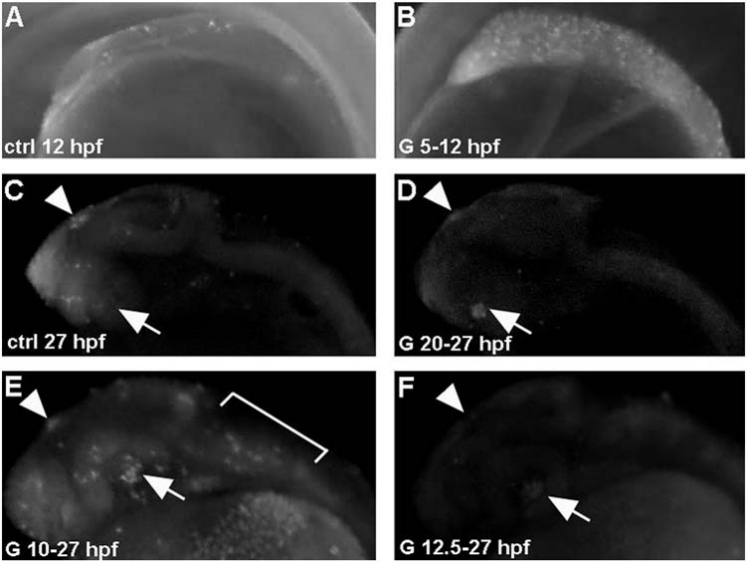
Genistein-induced apoptosis is developmental stage-dependent. Embryos were treated with 2.5 µM genistein for 7 hours starting at 5 hpf (B) or 20 hpf (D) and stained with acridine orange. A high number of apoptotic cells, compare with control (A), are detected in embryos treated from 5 hpf onwards. A similar localization (retina and epiphysis, arrow and arrowhead, respectively) and small number of apoptotic cells are detected in control (C) and genistein-treated embryos from 20 hpf onwards (D). (E) Treatment from 10 hpf onwards induces a strong apoptosis detected at 27 hpf (arrows), while a similar treatment started at 12.5 hpf did not induce apoptosis at 27 hpf (F).

In order to shed light into the developmental stage at which apoptosis can be detected in genistein treated embryos, we performed time course experiments. We thus treated embryos from 5 hpf onwards with 10 µM of genistein and we then detect apoptotic cells using acridine orange staining at different developmental time points. At 10 hpf (5 hours after the beginning of the treatment) no acridine orange positive staining was detected suggesting that at this time genistein does not yet induce apoptosis at these developmental stages (Table 1). At 12 hpf (7 hours after treatment), a few apoptotic cells were detected in control embryos but this number was much higher after genistein treatment (Table 1; Figure 2A and B). We observed the first apoptosis in control embryos in the hindbrain. Interestingly, the region in which genistein induced massive apoptosis, contains this area but extends further posteriorly in the anterior spinal cord. At 15 hpf the number of apoptotic cells in genistein treated embryos largely increases (Table 1) and apoptosis is detected in almost the entire CNS.

**Table 1:** Timing of the presence of apoptotic cells after 2.5 µM genistein treatment.

Time of	Control	Genistein 2.5 µM
**10 hpf**	No apoptotic cells	No apoptotic cells
**12 hpf**	Presence of few apoptotic cells in the brain	Presence of more apoptotic cells than in control (brain and spinal cord)
**15 hpf**	Presence of few apoptotic cells	Presence of a high number of apoptotic cells

These experiments reveal that apoptosis is detected as early as 7 hours after genistein exposure. This suggests that genistein is acting during a precise developmental window around 12 hpf. To further substantiate the existence of such a precise window we thus treated embryos with 2.5 µM of genistein at 5 hpf or 20 hpf and monitored apoptosis 7 hours after exposure (Figure 2A–D). Except in the lens and the epiphysis that are positive for acridine orange in control embryos (Figure 2C arrow and arrowhead, respectively), no apoptotic cells were observed on embryos treated at 20 hpf, while strong apoptosis is detected 7 hours after exposure on fish treated from 5 hpf onwards (compare Figure 2B and 2D). No apoptotic cells were observed later during embryonic development on embryos treated at 20 hpf (data not shown). This suggests that there is effectively a specific early developmental window of genistein sensitivity, during which exposure induces apoptosis.

Therefore, we decided to define more precisely the length of this developmental window by treating zebrafish embryos at different time points and assaying for apoptosis at 27 hpf (Figure 2E–F). In this experimental setup, we observed that apoptosis could be observed in embryos treated up to 10 hpf (Figure 2E, bracket), whereas no apoptosis is detected when treatment starts at 12.5 hpf (Figure 2F) or later (data not shown). This confirms the idea that genistein acts during a precise developmental window up to the beginning of somitogenesis.

Altogether these experiments suggest that genistein induces apoptosis during the end of gastrulation or in the early somitogenesis stages. Moreover, even if genistein induces effects that can still be detected at late developmental stages (up to 48 hpf), the exposure must start prior to 12.5 hpf to be effective.

### The genistein-induced apoptosis is ER-independent

**Figure 3:**
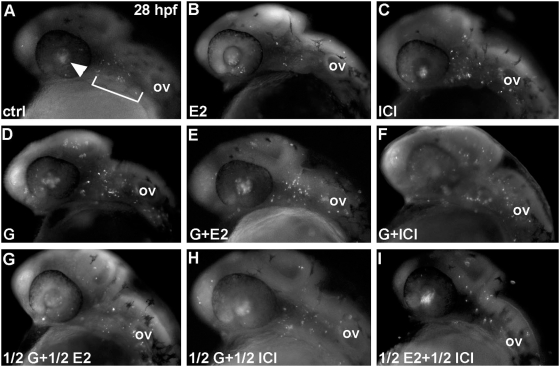
Genistein-induced apoptosis is estrogen receptor independent. (A,B) estradiol does not induce apoptosis. Embryos treated with 1 µM of E2 from 5 hpf onwards display the same pattern of apoptosis (retina and epiphysis) as controls (compare A and B). In contrast embryos treated with 1 µM ICI 182,780 exhibit an increased number of apoptotic cells in the hindbrain (C). Genistein induce an apoptosis at 5 mM as shown Figure 1 (D) and cotreatments with E2 (E) or ICI 182,780 (F) does not modify this effet. Effects of treatments with half dose (0.5 µM for E2 and ICI 182,780 and 2.5 µM for genistein) led to the same conclusions. See Supplementary Figure 2 for the quantitative analysis of these results.

Genistein is a well-known estrogen receptor agonist (see [16] for a review). We thus tested whether E2, the natural ligand of estrogen receptors, was able to induce a similar effect as genistein. Given that estradiol binds and activates the zebrafish ERs with affinities in the nanomolar range [48], [49], we treated embryos at 5 hpf with up to 1 µM of E2 (Figure 3B) and assayed the induced apoptosis with acridine orange staining at 28 hpf. As in the control (Figure 3A), very few acridine orange positive cells are detected suggesting that exogenous E2 does not induce apoptosis in early zebrafish embryos (Figure 3B). Figure S2 shows the quantitative analysis of these results after counting the number of apoptotic cells in embryos stained with acridine orange, using 40 embryos per point.

This result suggests that the apoptosis induced by genistein exposure may not be mediated through the estrogen signaling pathway. To test if a link exists between genistein-induced apoptosis and estrogen signaling we co-treated embryos with genistein and with ICI 182,780, a classical estrogen receptor antagonist [50]. ICI treatment alone induces apoptosis at concentration of 1 µM (Figure 3C), an effect seen in numerous studies using different model systems [51]. This apoptosis induced by ICI is effectively E2 dependant since it is reverted by co-treatment of ICI and E2. Indeed, as shown in Figure S2, lower panel cotreatments with E2 and ICI at a 1∶1 ratio very severely shut down the apoptotic effect induced by ICI. As shown above, genistein induces a strong apoptotic effect (Figure 3D; see Figure S2 for quantitative data and statistical significance) at a concentration higher than ICI (5 µM for Genistein vs 1 µM for ICI). A full dose of genistein (5 µM) combined with a full dose of E2 (1 µM) or a full dose of ICI 182,780 (1 µM) lead to a result similar to that of genistein treatment alone with no statistically different number of apoptotic cells present in each fish (Compare Figure 3D with 3E and 3F). These results lead to two conclusions: (i) genistein induces apoptosis in an ER independent manner as cotreatment with estrogen receptor agonist or an antagonist does not have any effect on the apoptosis induced by genistein; (ii) genistein does not synergise with ICI in terms of inducing apoptosis as the same number of apoptotic cells is observed with a genistein exposure alone or in combination with ICI 182,780 (see Discussion).

To better test if genistein has a synergistic effect with the estrogenic pathway we then co-treated embryos with half a dose of genistein (2.5 µM) and half a dose of E2 or ICI (0.5 µM). Again, we did not observe any synergistic effect between ICI and genistein comparable to a full dose of genistein (compare Figure 3D and 3H; see also Figure S2). As a control, a half dose of E2 and ICI showed as expected a very low number of apoptotic cells (Figure 3I). Of note treatment of zebrafish embryos with half-dose (2.5 µM) genistein and a full dose (1 µM) of ICI, in order to increase the ICI/Genistein ratio do not induce a significant effect on the genistein-induced apoptosis (not shown). We verified that the ICI compound we used is a potent zebrafish ER-antagonist both *in vivo* and *in vitro* (Table 2, see below). To check that the results we obtained were not specific of the type of antagonist used we also treated fish with tamoxifen (Figure S2). We observed that at 1 µM tamoxifen induce a modest increase in the number of apoptotic cells but that, as with ICI, co-treatement by genistein and tamoxifen does not alter, positively or negatively, the effect of genistein.

**Table 2:** Genistein elicit a transcriptional response *in vivo* using ERE reporter fish. Three independent experiments were performed.

Ligands	Fold induction
Ethanol	none
1 µM E2	X 655+/−50
2.5 µM Genistein	X 215+/−20
1 µM ICI	none
1 µM E2+10 µM ICI	none
2.5 µM Genistein+1 µM ICI	none

Taken together our results demonstrate that the action of genistein for apoptosis induction is estrogen-receptor independent.

### Genistein binds to and activates the three zebrafish ERs

The previous observation of a clear estrogen independent effect of genistein during zebrafish embryonic development led us to test if genistein is effectively able to induce an estrogenic response in zebrafish embryos.

**Figure 4:**
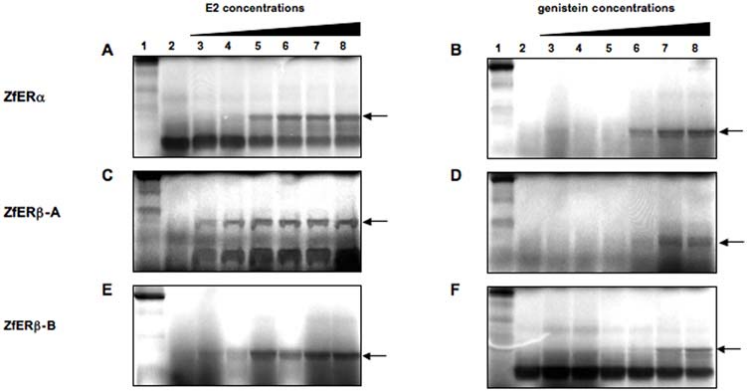
Genistein binds the three zebrafish ERs. Limited proteolysis assays of *in vitro* translated zebrafish ERα, ERβ-A and ERβ-B with E2 as ligand (A,C,E, respectively) and genistein as ligand (B,D,F, respectively). For each proteolysis panel, the first line represents the undigested protein, line 2 shows digestion of the receptor in the absence of ligand, lines 3 to 8 show digestion of the receptor in the presence of 10 fold increased concentrations of E2 and genistein from 10^−8^ M to 10^−3^ M.

The zebrafish genome contains three ERs: ERα, ERβ-A and ERβ-B ([48], [49]; see [52] for nomenclature and complete expression patterns). To test if genistein effectively binds to the zebrafish ERs, we used a limited proteolysis assay, which is a widely used method that allows testing whether a given ligand induces a conformational change of the receptor [53]. As seen in Figure 4, the three zebrafish estrogen receptor proteins are protected against trypsin degradation by E2 in a dose-dependent manner. We observed a faint protection starting at 1 µM of E2 (lane 5) for ERα, 0.01 µM of E2 (lane 3) for ERβ-A and 1 µM of E2 (lane 5) for ERβ-B and a plateau at 10 µM (lane 6), which is similar for all receptors. Thus, in this assay, ERβ-A appears to bind E2 at lower concentrations than ERα and ERβ-B. When we used genistein, we observed that all receptors are protected against trypsin degradation and that the protection starts at 10 µM (lane 6) for ERα and ERβ-A and 100 µM (lane 7) for ERβ-B. We therefore conclude that genistein is able to bind to all three zebrafish estrogen receptors with a comparable range of affinity. Even though limited proteolysis assay cannot be used to define a Kd value it seems clear from these experiments that the affinity of the three zebrafish ERs for their natural ligand E2 is much higher than it is for genistein. This clearly validates the range of concentration used previously (see Figure 3) to measure the combined effect of genistein and E2 on apoptosis.

**Figure 5:**
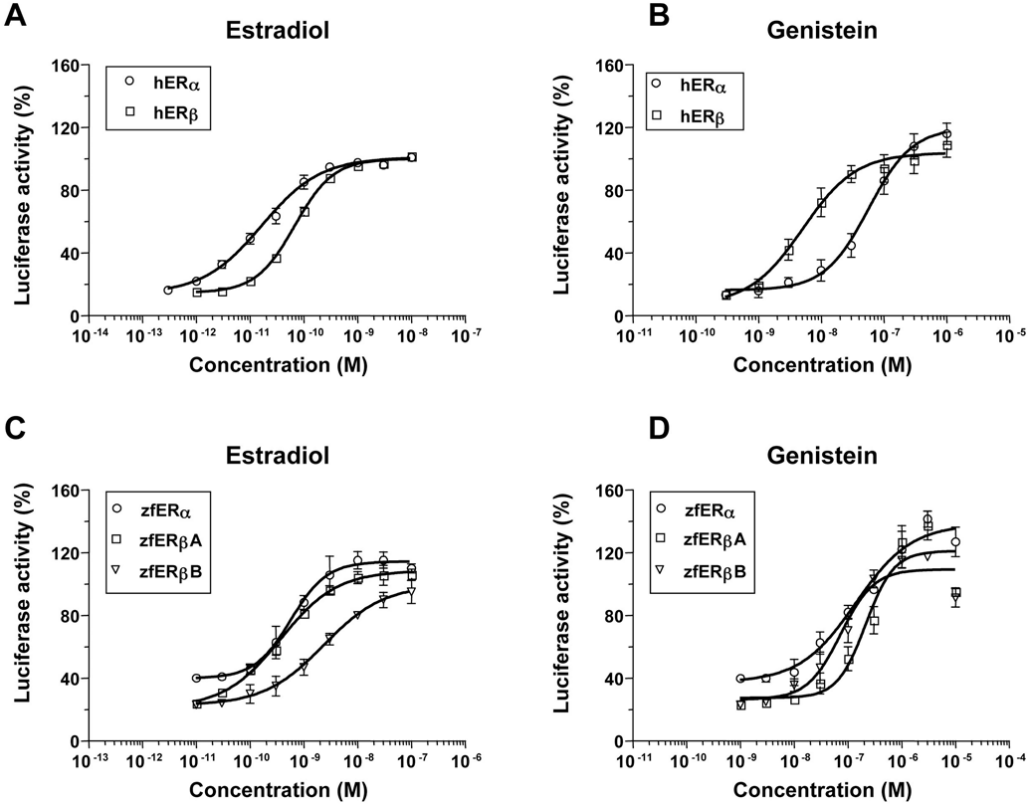
Genistein activates the three zebrafish ERs. In transient transfection (A–E), genistein is able to modulate the transcriptional activity of the three zebrafish estrogen receptors in a less effective way than their natural ligand E2. We did not observe a difference of genistein-induced activity between zebrafish ERα and ERβ as there is for human ERα and ERβ (compare A with C).

Similarly, we observed that genistein is able to elicit a transcriptional activation of the ERs in transient transfection assays in Hela cells, which do not have endogenous estrogen receptors. These cells were transiently transfected with a luciferase reporter gene under the control of a classical ERE element (ERE-β Globin-Luc), together with expression vectors encoding the zebrafish estrogen receptors genes ERα, ERβ-A and ERβ-B. E2 induces a clear activation of zebrafish ERs starting at 10^−10^ M for ERβ-A and 5×10^−9^ M for ERα and ERβ-A (Figure 5C) in accordance with previously published data [48], [49]. Genistein has similar effects on the three receptors with a very faint activation detectable at 10^−8^ M and a maximal effect at 10^−6^ (Figure 5D). In contrast, we observed that, as previously published on human ERs, genistein has a clear selectivity in favor of ERβ since ERβ is activated at a 10 fold lower concentration (10^−9^ M) than ERα (10^−8^ M) (compare Figure 5B with D) [13], [18], [19], [23], [54]. Taken together our results clearly demonstrate that *in vitro* genistein has the capacity to bind and activate all zebrafish ERs in a comparable manner.

**Figure 6:**
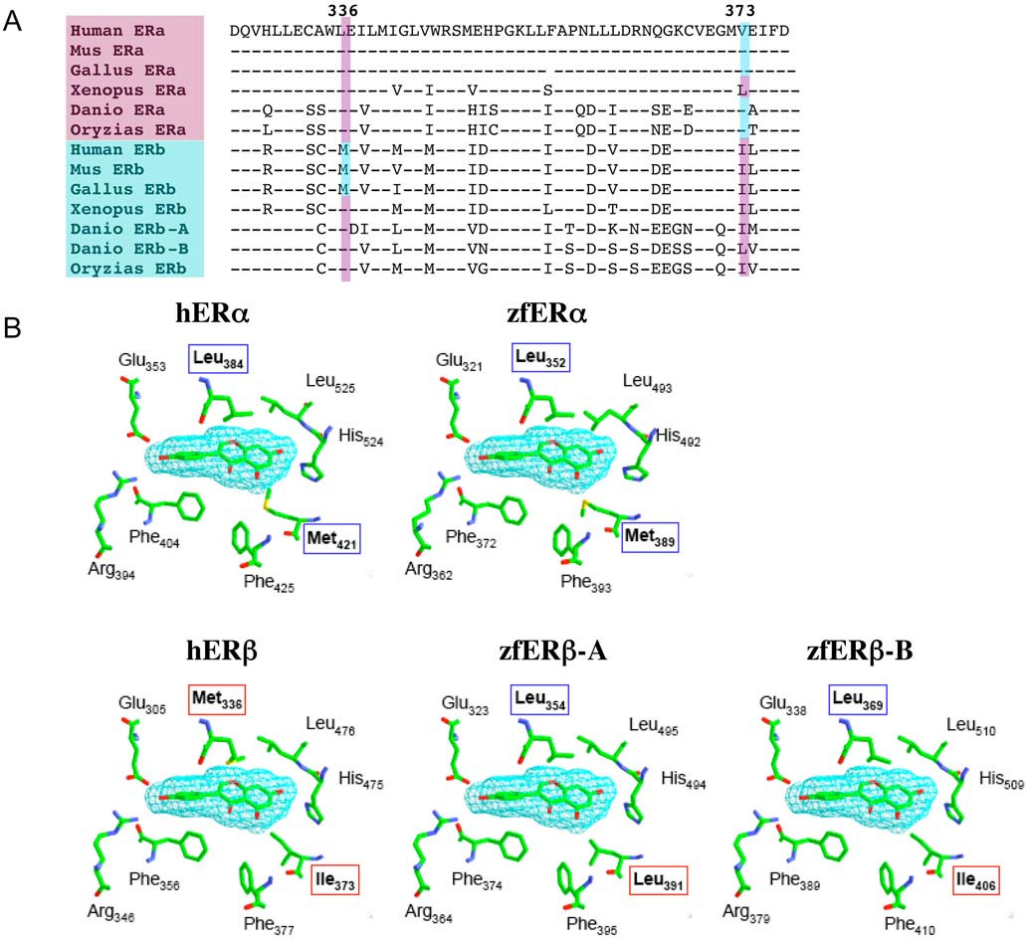
(A) Alignment of a part of the ligand binding domain of several vertebrate ERs. The two main amino acids involved in the binding of genistein are underlined. Blue for human ERβ-like amino acids and pink for human ERα-like amino acids. (B) modelling of the ligand binding pocket of estrogen receptors in presence of genistein. The sequences used for the modelling were from human (hERα and hERβ) or from zebrafish (zfERα, zfERβ-A, zfERβ-B). The α-type amino acids are boxed in blue whereas the β-type amino acids are boxed in red. The non variable amino acids are not boxed. Numbering refers to each receptor.

In humans, the structural basis of the selectivity of genistein for ERβ versus ERα has been linked to two critical positions in the ligand binding pocket, Met 336 and Ile 373, which are replaced by a Leu and a Met respectively in ERα ([23]; see Figure 6A). In the zebrafish ERs, we observed that all ERs have a leucine residue at the position homologous to Met 336 in the human ERβ meaning that all zebrafish ERs are “ERα-like” for this residue. For the other position (Ile 373 in ERβ), the two zebrafish ERβs have an Ile like human ERβ, while the unique ERα has a Met, like its human ERα ortholog. All together these results show that one of the two crucial amino acids directly involved in the binding of genistein to ER in all zebrafish sequences has a signature of an ERα type. Exactly the same situation is found, when analyzing the sequences of ERs from another fish model, the medaka *Oryzias latipes*. To visualize the impact of these substitutions in the fish ERs we performed a 3D modeling analysis of the ligand binding domains from zebrafish ERs (Figure 6B). As expected after primary structure analysis, the residues lining the ligand binding pocket of human ERs and zebrafish ERs are very conserved with the few pointed exceptions and are organized in a similar 3D configuration. However, the slight change observed in Met 389 positioning in zebrafish ERα, and the substitution of Met 336 in human ERβ by a Leu residue in zebrafish ERβ (at position 354 and 369 in zebrafish ERβ-A and zebrafish ERβ-B respectively), are likely to equalize the relative affinity of genistein for zebrafish ERs. Taken together these data suggest that the lack of selectivity of genistein activation of zebrafish ERs may be explained by these specific amino acid substitutions and slight change in the relative positioning of certain residues.

### Genistein elicits an estrogenic response *in vivo*

To demonstrate that genistein can elicit a transcriptional response *in vivo* we used a transgenic reporter zebrafish line that contains a luciferase gene under the control of an ERE linked to a minimal promoter (3×ERE-TATA-Luc) [55], [56]. We performed this assay in juvenile fishes (3 months old) since, in our hands, E2 does not induce a detectable luciferase response using these transgenic fishes at embryonic stages (data not shown). The transgenic ERE-Luc fish were treated with genistein at 2,5 µM in absence or presence of ICI (1 mM). The Luc activity was then measured from pooled pairs of juveniles (Table 2) and was at background levels in non-treated fishes (not shown). In presence of 1 µM E2, we observed a very strong (620×) induction of Luc activity, whereas, as expected, ICI 182,780 does not induce any activation and completely abolishes E2 induced activity. In presence of 2,5 µM genistein, Luc activity is well induced (215 fold), but no induction is detected when we co-treated with 1 µM of ICI (Table 2) clearly suggesting that this effect is ER-dependent. We conclude from these results that genistein can activate the estrogen pathway *in vivo* through a direct binding to the estrogen receptors.

### Genistein regulates *aromatase-B* expression in an estrogen dependent manner

**Figure 7:**
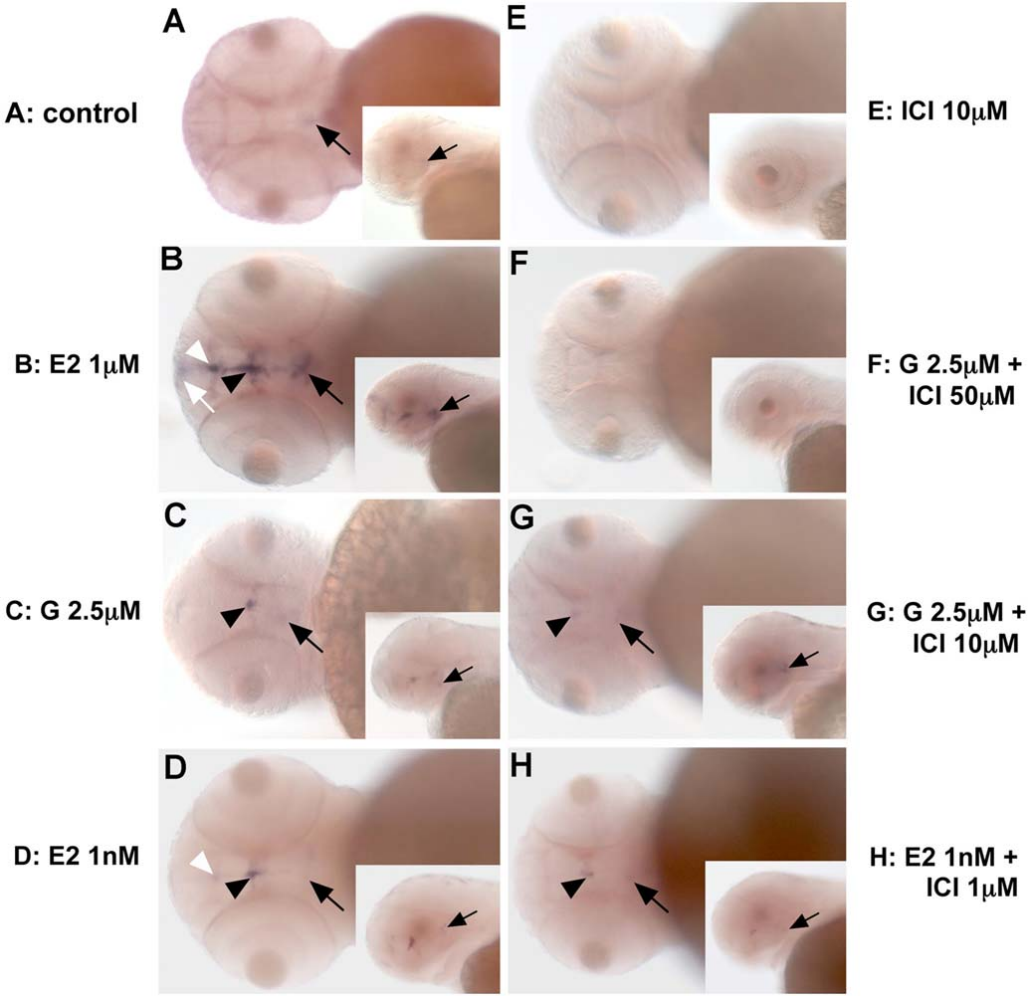
Genistein regulates *aromatase-B* expression in the brain in an estrogen receptor-dependent manner. *In toto in situ* hybridization showing the expression of *aromatase-B* in the zebrafish brain at 48 hpf. (As) Control embryo with weak expression detected in mediobasal hypothalamus (MBH) (arrowheads). (Bs) After genistein exposure, expression is observed in MBH (arrowheads) and in the preoptic area (POA) (arrows). ER antagonists, like ICI, do not induce *aromatase-B* expression (Cs). The weak staining observed in the MBH in controls is no longer detected in ICI-treated embryos. Cotreatment of genistein and ICI abolishes *aromatase-B* expression (Ds), but a low dose of ICI does not totally block genistein-induced *aromatase-B* expression (compare Ds with Es). E2 at high concentrations induces expression of *aromatase-B* in the MBH (arrowhead), POA (arrow) and in the telencephalon (Tel). When a lower concentration of E2 (1 nM) is used, expression is no longer detected in the anterior brain (Tel) but remains in the MBH (arrowhead) and POA (arrow) as after genistein exposure (compare Gs and Bs). As for genistein, cotreatment of ICI with low concentrations of E2 strongly reduces *aromatase-B* expression (Hs).

Given that the estrogenic effect of genistein *in vivo* described above was obtained in larvae, we studied if genistein can elicit a transcriptional response on an endogenous gene with embryonic expression. We thus studied if genistein was able to induce the expression of the *aromatase* (*CYP19*) genes, classical ER target genes *in vivo* (reviewed in [57]). E2 production via aromatase is crucial for development, growth and differentiation of the brain and has been well characterized in zebrafish [58]. Indeed, the teleost fish brain is characterized by an important aromatase activity due to the expression of a brain-specific *aromatase-B* gene (*aro-B*) [59]. The expression of this gene is upregulated by E2 and recent studies indicate that the *aro-B* gene can be used as a sensitive marker of the effects of xenoestrogens on the central nervous system during embryogenesis [60] and in zebrafish juveniles [61]. Whole mount *in situ* hybridizations were performed on 48 hpf embryos using *aromatase-A* and *-B* probes. We were not able to detect *aromatase-A* transcripts by *in situ* during embryogenesis but we could detect embryonic expression of this gene by RT-PCR (data not shown). In contrast, we observed a faint signal with the *aro-B* probe in the mediobasal hypothalamus (MBH) (arrow Figure 7A). Therefore, we focused on the *aromatase-B* expression at 48 hpf, in order to compare the effects in terms of gene expression in response to genistein and E2 treatments.

In 48 hpf embryos, after E2 treatment (1 µM) from 5 to 48 hpf, *aromatase-B* transcripts were detected in the olfactory area (OA) (white arrow), the telencephalon (TEL) (white arrowhead), the preoptic area (POA) (black arrowhead) and strongly in the MBH (black arrow) (Figure 7B) in accordance with recently published data [62]. In embryos treated with genistein (2,5 µM), we observe clear staining in the PAO and in the MBH (Figure 7C, arrowhead and arrow, respectively), but no signal can be detected in the telencephalon or in the olfactory area. Interestingly, this induced expression pattern for *aro-B* resembles what is observed when a low dose of E2 is applied to the developing embryo. Indeed, when treated with 1 nM of E2 *aromatase-B* transcripts are detected in the POA and the MBH (Figure 7D) in a pattern similar to the one observed when the embryos are exposed to 2.5 µM of genistein (compare Figure 7C and 7D). In 1 nM E2 exposed embryos, a faint staining of *aro-B* is detected in the POA (white arrowhead, Figure 7D), which is absent in genistein treated embryos. We therefore conclude that genistein mimics a weak effect of E2 in term of *aromatase-B* expression in the brain.

To confirm that the genistein-induced *aro-B* expression is ER dependent, we co-treated embryos with the ER antagonist ICI 182,780 and with genistein. As expected for an ER-mediated response, ICI treatment alone or combined with genistein totally abolished *aro-B* induction in a dose-dependent manner (Figure 7E–G). Furthermore, co-treatment of zebrafish embryo with E2 and ICI can severely reduce expression of the aro-B gene (Figure 7H). These data confirm that genistein can elicit a clear estrogenic response *in vivo* in zebrafish embryos. This is in accordance with the observation that these embryos express the estrogen receptors as well as several transcriptional coactivators from early developmental stages through larval stages [48], [52].

## Discussion

In this paper, we report that genistein induces two distinct responses in zebrafish embryos: (i) it specifically induces apoptosis during early embryogenesis in an estrogen receptor-independent manner. Importantly, we observed that this genistein-induced apoptosis is linked to a specific developmental process since exposure of genistein later in development does not lead to the same effect; (ii) it induces a clear estrogenic response *in vivo* by regulating the *aromatase-B* expression, a classical ER-target gene *in vivo* in an ER-dependent manner. In addition, we show that genistein is a potent zebrafish ER agonist both *in vitro* and *in vivo*. Our results thus highlight the multiplicity of possible actions of phytoestrogens, such as genistein. This suggests that the use of standardized endpoints to study the effect of a given compound, even when this compound has well known targets, may carry the risk of overlooking interesting effects of this compound.

### Genistein exposure induces apoptosis during zebrafish embryogenesis

Few studies have dealt with the effect induced by an isoflavone like genistein during embryonic development. Therefore, little is known about the developmental effects induced by exposure of this compound during vertebrate embryonic development. In quail embryos, for example, administration of genistein after three days of incubation leads to a decrease of copulatory behavior at puberty [63], but the possible effects during embryogenesis have not been assessed. The zebrafish is an excellent model for such a study, given its transparency and the fact that its development is well known. Using the zebrafish embryo, we showed a clear *in vivo* developmental effect induced by genistein: a strong apoptosis mainly located in the hindbrain and the anterior spinal cord. Interestingly, genistein also induces apoptosis in the brain, when adult Sprague-Dawley rats where given up to 20 mg/day genistein [64]. The same study has shown that in rat brain tissue homogenates as well as in primary cultures of rat cortical neurons high concentrations of genistein decrease the expression of the 32 kDA *caspase 3* precursor and increase the levels of the cleaved active form of this caspase that is well known to be instrumental in the apoptosis pathway. One putative scenario for genistein induction of apoptosis in the CNS could therefore be by stimulating the cleavage of the Caspase 3 protein. It therefore would be of interest to monitor the balance between cleaved and uncleaved Caspase 3 protein in the developing brain of zebrafish embryos exposed to genistein. It has to be noted, however, that genistein can induce apoptosis by modifying either the expression level or the phosphorylation status of several actors of the apoptotic cascade as has recently been shown using human neuroblastoma cells [65]. These data suggest that the precise manner, by which genistein induces apoptosis remains to be resolved.

Recently another study has suggested that genistein can induce early developmental defects. Indeed, using mouse blastocysts, Chan et al., have shown that genistein treatment induces apoptosis, decreases cell number and delays early post implantation development [66]. Even more interestingly these authors also reported that genistein treatment increases early-stage death *in vitro* suggesting that it negatively affects early mouse embryonic development *in vivo* by inducing cell apoptosis and by inhibiting cell proliferation [66]. Interestingly in this study, treatment with daidzein, another soy isoflavone, does not have an effect similar to the one we observe in zebrafish embryos.

All these data clearly suggest a need for more studies on the apoptotic effect of genistein and related isoflavonoids during development as well as in adults. It will be particularly interesting to address, whether the reported chemopreventive effect of genistein on breast cancer and other malignancies can be linked to its apoptotic effect. It is important to note that the level of genistein needed in our study to obtain an effect (2.5 µM) is only 10 fold higher to the levels reported in human plasma (up to 300 nM) [67].

### The apoptotic effect of genistein is ERs independent

It has been demonstrated that isoflavones can also exert biological effects independent of their phytoestrogenic activities [68]. In the present study we report an effect of genistein in inducing apoptosis that is apparently independent of the estrogen receptors. Indeed, estrogen exposure does not have an effect that is similar to genistein treatments and we did not observe a block of genistein-induced apoptosis when we cotreated fish with classical ER antagonists (either ICI 182,780 or tamoxifen), and genistein. Given that ICI 182,780 will occupy the ERs expressed during early zebrafish embryogenesis, its presence in addition to genistein should lead to a decrease of genistein action, that is a decrease in the number of apoptotic cells. This is exactly the effect we observe when clear estrogenic actions of genistein are studied that is the luciferase activity in the ERE-Luc reporter transgenic fish and the *aromatase-B* expression in zebrafish brain. In contrast, suing several ratios between ICI 182,780 and genistein we did not see any inhibitory (nor synergistic) effects between both compounds. All these data thus suggest that genistein induces apoptosis through another target than estrogen receptors.

Two main alternative targets for genistein have been described. It has been shown, two decades ago, that genistein is a direct potent inhibitor of tyrosine-specific tyrosine kinases, such as EGF receptor or the proto-oncogenes *src* and *fes* [27]. More recently genistein was shown to bind to transthyretin and to have a beneficial effect as an inhibitor of transthyretin amyloidosis [26]. Several studies have also reported that both tyrosine-specific tyrosine kinase of the EGF receptor and transthyretin play a role in apoptosis. It is possible that the high number of apoptotic cells observed in zebrafish embryos after exposure to genistein might be due to a deregulation of at least one of these genes.

Recently the genes responding in male zebrafish liver to the exposure to 17 β -estradiol, and genistein were identified through a microarray analysis [69]. Interestingly these data reveal that fish exposed to 17 β-estradiol and genistein have similarities in their gene expression patterns and genistein was found to regulate several genes involved in cell cycle-regulation. These observations are fully consistent with estrogen receptor-independent and -dependent effects of genistein that we describe here through developmental analysis.

### No ERα/β selectivity in fish for genistein

In this paper, we demonstrate that genistein binds and activates the three zebrafish ERs (Figure 4 and 5). Moreover we show that despite to what is happening in humans, where there is a considerably weaker affinity of genistein for ERα [13], [18], [19], [23], [54], there is no major difference in the binding and transactivation capacity of the three different zebrafish ERs (Figure 5C, D). According to Manas et al. 2004, two key amino acids involved in genistein binding differ between human ERα and making the ERβ more selective to genistein [23]. We observed that one of these amino acids (the position homologous to Met 336 in human ERβ) is mutated in fish ERβs making them similar to ERα (Figure 6A). For the second amino acid, the α/β difference found in the human receptors is maintained in fish: Met for ERα and Ile for ERβ (Figure 6A). Therefore, a single amino acid difference is apparently not sufficient to have α/β difference of affinity for genistein binding. In addition, comparative analysis of the interaction between genistein and the residues that line the ligand binding pocket clearly indicates that the substitution of human ERβ Met 336 by Leu (at position 354 and 369 in zebrafish ERβ−A and ERβ−B, respectively) is likely to decrease the affinity of zebrafish ERβs for genistein as compared to human ERβ (Figure 6B). This observation is in agreement with the measured EC_5O_ in reporter assay (Figure 5) that show a one log increase for zebrafish ERβs as compared to human ERβ. Interestingly, frogs display the same pattern of amino acids as fish for these two particular amino acids and differences to this trend are only observed at the level of the amniotes (Figure 6A). These data suggest that variations in the ligand binding pocket may have occurred during vertebrate evolution that might explain species-specific patterns in the effects elicited by a given compound. A similar example, linked to a pharmacological difference in the binding of synthetic compounds, has recently been observed for retinoic acid receptors in fish and *Xenopus* versus amniotes [53]. In the case of ERs this species difference is interesting and may have important functional consequences in the precise pharmacology of the receptors in terms of the response to natural compounds, such as phytoestrogens, and synthetic ones, such as endocrine disruptors. Clearly our data prompt for a careful analysis of the transcriptional patterns generated by a wide variety of compounds in fish and mammalian ERs.

## Materials and Methods

### Fish stocks

Zebrafish of the Konstanz wild type strain and the AB/TU wild type strain were reared at 28.5°C and staged as previously described [45]. No differences in the effects of genistein on these two strains were observed. The development of endogenous pigments was inhibited by exposing embryos to 1-phenyl-2-thiourea (PTU) at a final concentration of 0.2 mM. We specifically checked that this PTU treatment does not have any effect on apoptosis *in vivo*.

### Treatment of zebrafish embryos

Embryos were incubated with various concentrations of genistein (Sigma) (from 0.1 µM to 17.5 µM), 17β-estradiol (E2) (from 1 nM to 0.1 µM), ICI 182,780 (from 0.5 µM to 50 µM), genistin (Calbiochem), apigenin (Sigma) and daidzein (Sigma) at 10 µM diluted in embryo medium from a 10^−1^ M stock solution in ethanol from 5 hpf onwards unless specified otherwise. As controls, wild-type embryos were treated with equivalent amount of ethanol.

### Whole-mount in situ hybridization and photography

Whole-mount *in situ* hybridizations were performed as previously described [70]. Images were processed using Zeiss Stereomicroscope LUMAR with epifluorescence and Adobe Photoshop software

### Cloning of the aromatase-B probe

Zebrafish *aromatase-B* probe [62] was amplified by PCR using the following primers (forward: 5′-GACGGATCCAGCATGTGfGTAAAGGATGCGG-3′; reverse: 5′-CCGCTCGAGGAGACCTGGACCTGTAAGAG-3′) and cloned into the pCRII-TOPO vector (Invitrogen).

### Acridine orange staining

Live embryos were stained for apoptotic cells with the vital dye acridine orange (Sigma) that permeates acidic lysosomal vesicles and becomes fluorescent, thus marking cells dying by apoptosis. The stock solution (5 mg/ml in egg water, 300×) was diluted to 1× concentration and dechorionated embryos were bathed in this solution for 20 minutes in the dark. Embryos were washed in embryo medium and analyzed under a fluorescence microscope. 40 embryos per points were manually counted in blind experiments for the quantitative data (see Sup. Figure 2)

### TUNEL assays

Embryos were fixed in 4% paraformaldehyde in PBS for 4 hours at room temperature, washed twice in PBS-Tween 0.1% and stored in methanol at −20°C, following progressive dehydratation. Assays were performed using *in situ* cell death detection kit (POD, Roche) as described by the manufacturers. Cell death was detected either using the peroxidase reaction or by directly detecting the incorporated FITC-labeled nucleotides using a fluorescent microscope [47].

### Transient transfections

HeLa cells were maintained at 37°C in 5% CO_2_ in Dulbecco's modified Eagle medium (DMEM) F12 with phenol red, supplemented with 5% FCS and 1% antibiotic (penicillin/streptomycin). Transfections were performed with Jet-PEI according to the manufacturer's recommendations (Ozyme, Saint Quentin-en-yvelines, France) and using 0.1 µg of the pSG5 expression vector for the ERα, ERβ-A and ERβ-B, 0.4 µg of the estrogen-dependent luciferase reporter construct (ERE-β-globin-Luc SV-Neo) and 0.2 µg of the internal reference reporter plasmid per well (CMV-Gal). After overnight incubation, the medium was removed and the cells were placed into fresh medium supplemented with control vehicle or compounds. After 24 h, cells were harvested and assayed for luciferase and b-galactosidase activities as previously described [71].

### Luciferase assay in transiently transfected cells

Cell lysates were prepared as recommended by Promega. Briefly, cells were washed twice with 1 ml of PBS and lysed with 0,4 ml of lysis buffer [25 mM Tris-phosphate (pH 7,8), 2 mM EDTA, 10% glycerol and 1% Triton X-100] for 10 min. Cell lysate (100 µl) was transferred in a 96 well plate white opaque tissue culture plates (Greiner Cellstar, D. Dutscher) and maintained in 6% DCC-FCS. Genistein was added 8 hours later and cells incubated with compound for 16 hours. Luminescence was detected after injection of 100 µl of luciferase detection buffer [2 mM Tricine (pH 7,8), 1.07 mM (MgCO3) 4Mg(OH)2, 2.67 mM MgSO4, 0.2 mM EDTA, 0.53 mM ATP, 0.27 mM CoA and 0.48 mM luciferin] and the 96-well plate was then introduced into a microplate luminometer (Microbeta) and intact living cell luminescence measured for two seconds. Arbitrary units in transient transfection experiments represent a ratio of luminescence to β-galactosidase activity used as transfection control.

### Limited proteolysis assay

The assays were performed as described in [52]. Genistein was incubated with the protein at different concentrations (from 10 nM to 1 mM). After incubation SDS-PAGE was performed using a 12% gel. Electrophoresis was carried out using a BioRad gel apparatus at 170 V for 50 minutes and the polyacrylamide gel was visualized by autoradiography.

### *In vivo* luciferase assay

We used the transgenic ERE-Luc fish line described by [54], [55]. Male fish with a weight of 300 mg (three to six months old) were exposed for 48 hours to one ligand or to a mixture of two ligands in glass aquaria (E2, ICI 182,780 and/or genistein). Ligands were dissolved in ethanol (or in DMSO) at concentrations of 10^−1^ M or 10^−3^ M and titrated to final concentrations not exceeding a solvent concentration of 0.01%. Single fish were anaesthetized (0.6 mM Tricain methanesulfonate) and transferred to an Eppendorf tube. Then, 135 ml of ice cold Triton-lysis buffer (1% triton-X-100; 15 mM MgSO_4_; 4 mM EGTA pH 7.0; 35 mM glycylglycine pH 7.8; 1 mM DTT) was added to each tube following homogenization using a micropestle. After centrifugation the supernatant was transferred to a new tube and extracts were measured in luminometric reporter gene assays carried out in duplicates in a Microplate Luminometer (Anthos). Light units from extracts of ligand-exposed fish and from non-exposed fish were used to calculate fold inductions.

### Bioinformatics

Partial sequences of estrogen receptor genes from various vertebrates were aligned using the ClustalW software available on the web (http://bioinfo.hku.hk/services/analyseq/cgi-bin/clustalw_in.pl).

### Construction of 3D models

3D structures of ERα, ERβ-A and ERβ-B of *Danio rerio* were modelled on the human ERα (1X7R) and ERβ (1X7J) using the web-based Swiss-Model server. The figure was prepared using the Swiss-PDB Viewer software (v3.9).

## Supporting Information

Figure S1 Detection of apoptosis in genistein treated fishes using TUNEL assays, a direct method for the presence of fragmented DNA (Lawry 2004). Fishes were treated at 5 hpf and labeled at 24 hpf with the indicated concentration of genistein. We observe a dose-dependent activation of apoptosis starting at 0.5 µM.
(0.79 MB TIF)

Figure S2 Genistein-induced apoptosis is estrogen receptor independent. Fish were treated at 5 hpf by the various compounds at the indicated concentration and apoptosis was monitored by acridine orange staining at 27 hpf. The number of apoptotic cell per fish was counted. Batch of 20 fishes were treated by the indicated compounds and the number of apoptotic cells per fish was counted after acridine orange staining. The experiment was done twice, thus a total of 40 fishes per point was analyzed. (Upper panel) Effect of ER agonists and antagonists on genistein induced apoptosis. G: Genistein; E2: 17β-estradiol; ICI: ICI 182,780; Tamox: Tamoxifen. It is interesting to note that neither E2 nor ICI or Tamoxifen alter the magnitude of the apoptotic effect elicited by genistein. (Lower panel) The apoptotic effect elicited by ICI treatment is reverted by a cotreatment with E2 suggesting it is ER-mediated. The statistical analysis shows the result of a t-test with a Bonferroni correction (***: p value<0.005). In the upper panel, we compare the number of apoptotic cells in each indicated column with the number of apoptotic cells present after a treatment at 5 µM genistein.
(0.28 MB TIF)
