# Supplementary figures and images for: The Phytoestrogen Genistein Affects Zebrafish Development through Two Different Pathways
Source: PLoS One. 2009 Mar 25;4(3):e4935. doi: 10.1371/journal.pone.0004935 (PMC2655710; doi:10.1371/journal.pone.0004935)

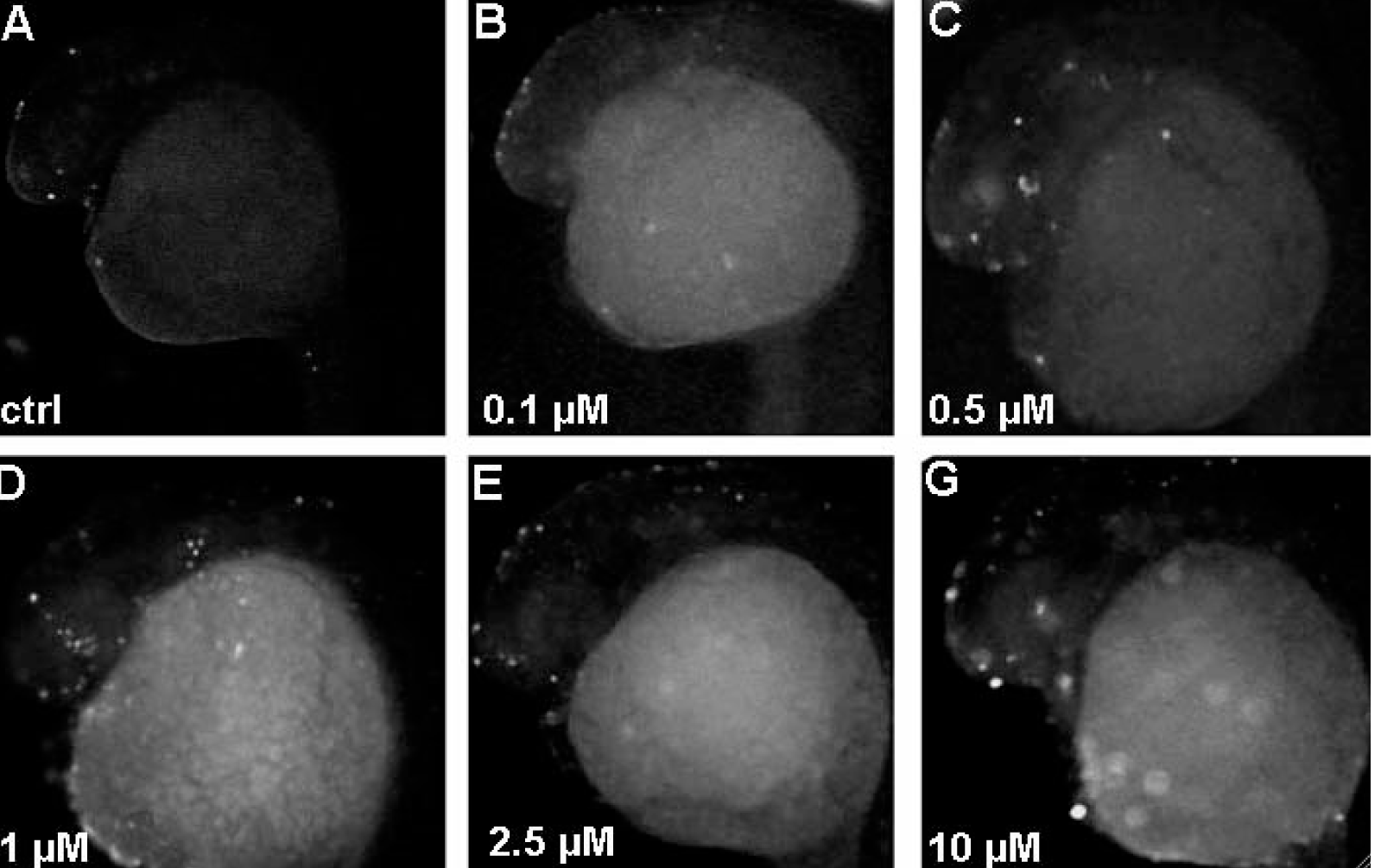

Supplement: Figure S1 — Detection of apoptosis in genistein treated fishes using TUNEL assays, a direct method for the presence of fragmented DNA (Lawry 2004). Fishes were treated at 5 hpf and labeled at 24 hpf with the indicated concentration of genistein. We observe a dose-dependent activation of apoptosis starting at 0.5 µM. (0.79 MB TIF) [file pone.0004935.s001.tif]

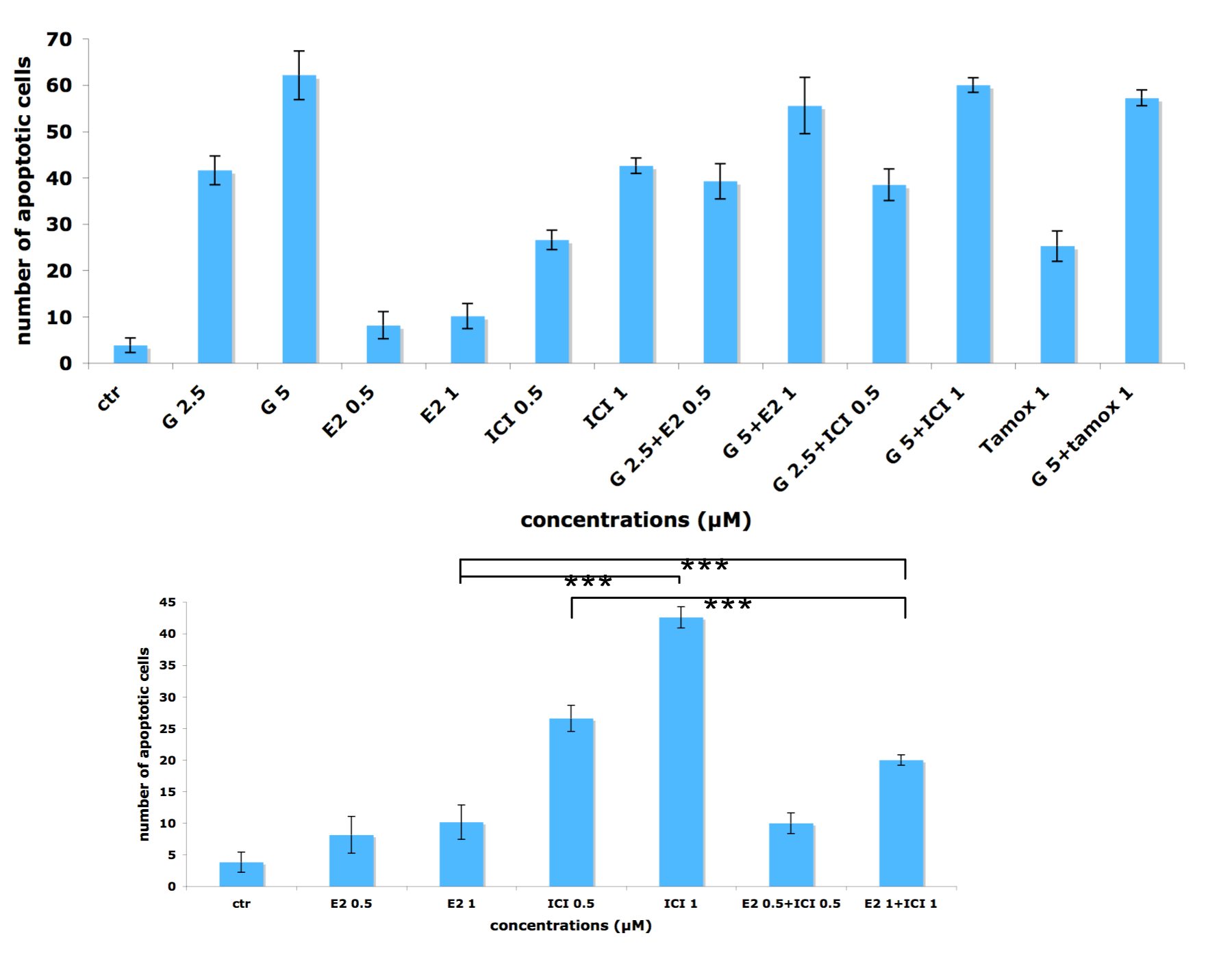

Supplement: Figure S2 — Genistein-induced apoptosis is estrogen receptor independent. Fish were treated at 5 hpf by the various compounds at the indicated concentration and apoptosis was monitored by acridine orange staining at 27 hpf. The number of apoptotic cell per fish was counted. Batch of 20 fishes were treated by the indicated compounds and the number of apoptotic cells per fish was counted after acridine orange staining. The experiment was done twice, thus a total of 40 fishes per point was analyzed. (Upper panel) Effect of ER agonists and antagonists on genistein induced apoptosis. G: Genistein; E2: 17β-estradiol; ICI: ICI 182,780; Tamox: Tamoxifen. It is interesting to note that neither E2 nor ICI or Tamoxifen alter the magnitude of the apoptotic effect elicited by genistein. (Lower panel) The apoptotic effect elicited by ICI treatment is reverted by a cotreatment with E2 suggesting it is ER-mediated. The statistical analysis shows the result of a t-test with a Bonferroni correction (***: p value<0.005). In the upper panel, we compare the number of apoptotic cells in each indicated column with the number of apoptotic cells present after a treatment at 5 µM genistein. (0.28 MB TIF) [file pone.0004935.s002.tif]
